# Supplementary material for: Chemical Profiles, In Vitro Antioxidant and Antifungal Activity of Four Different Lavandula angustifolia L. EOs
Source: Molecules. 2023 Jan 2;28(1):392. doi: 10.3390/molecules28010392 (PMC9822278; doi:10.3390/molecules28010392)
Supplement: Supplementary file 1 [file molecules-28-00392-s001.zip › molecules-2097691-supplementary.pdf]

Supplementary Materials

Chemical Profiles, In Vitro Antioxidant and Antifungal Activity of Four Different *Lavandula angustifolia* L. EOs

Claudio Caprari, Francesca Fantasma, Pamela Monaco, Fabio Divino, Maria Iorizzi, Giancarlo Ranalli, Fausto Fasano and Gabriella Saviano *

Department of Bioscience and Territory, University of Molise, C.da Fonte Lappone snc, 86090 Pesche, Italy
* Correspondence: saviano@unimol.it; Tel.: +39-0874404117

Table S1. Statistical analysis: ANOVA test and post hoc Duncan test.

N.	Compound	ANOVA test	Duncan test - groups			
		p-values	LaCC	LaPE	LaPS	LaPRV
1	Tricyclene	<0.01	c	b	b	a
2	*α*-Thujene	<0.01	c	b	c	a
3	*α*-Pinene	<0.01	d	a	c	b
4	Camphene	<0.01	d	b	c	a
5	Sabinene	<0.01	c	a	c	b
6	*β*-Pinene	<0.01	c	a	c	b
7	1-Octen-3-ol	<0.01	c	b	c	a
8	3-Octanone	<0.01	a	c	bc	b
9	Myrcene	<0.01	b	b	a	c
10	Butyl butanoate	<0.01	a	b	b	b
11	*α*-Phellandrene	<0.01	c	a	b	a
12	3-Carene	<0.01	c	a	b	a
13	*α*-Terpinene	<0.01	b	a	b	a
14	3-Undecen-1-yne	<0.01	a	b	a	b
15	*p*-Cymene	<0.01	a	c	d	b
16	Limonene	<0.01	c	a	b	a
17	1,8-Cineole	<0.01	d	a	c	b
18	(*Z*)-*β*-Ocimene	<0.01	a	a	b	a
19	(*E*)-*β*-Ocimene	<0.01	a	c	d	b
20	*γ*-Terpinene	<0.01	b	a	b	a
21	*cis*-Sabinene hydrate	<0.01	b	a	c	b
22	U	<0.01	a	b	c	b
23	Terpinolene	<0.01	d	a	c	b
24	Linalool	<0.01	b	c	d	a
25	Octen-3-yl acetate	<0.01	a	c	b	c
26	allo-Ocimene	NS	-	-	-	-
27	*trans*-Pinocarveol	<0.01	a	b	b	b
28	Camphor	<0.01	d	b	a	c
29	Borneol	<0.01	d	b	c	a
30	Lavandulol	<0.01	a	b	b	c
31	Terpinen-4-ol	<0.01	c	b	d	a
32	*α*-Terpineol	<0.01	c	b	a	c
33	Hexyl butanoate	<0.01	c	a	d	b
34	Estragole (Metil chavicol)	NS	-	-	-	-

35	Isobornyl formate	<0.01	c	b	c	a
36	Nerol	<0.01	b	c	a	c
37	Hexyl-2-metil butyrate	<0.01	d	a	c	b
38	Cumin aldehyde	<0.01	a	a	b	b
39	Hexyl isovalerate	<0.01	d	b	a	c
40	Linalyl acetate	<0.01	b	d	a	c
41	Bornyl acetate	<0.01	c	b	a	c
42	Lavandulyl acetate	<0.01	a	d	b	c
43	Hexyl tiglate	<0.01	d	a	c	b
44	Eugenol	<0.01	a	b	b	b
45	Neryl acetate	<0.01	b	c	a	c
46	Copaene	<0.01	a	b	b	b
47	Daucene	<0.01	c	b	a	c
48	β-Bourbonene	<0.01	a	b	b	b
49	*trans*-Myrtanol acetate	<0.01	b	c	a	c
50	Hexyl hexanoate	<0.01	b	a	c	b
51	7-epi-Sesquithujene	<0.01	c	a	c	b
52	Sesquithujene	<0.01	c	a	b	a
53	Longifolene	<0.01	b	a	b	b
54	(*E*)-Caryophyllene	<0.01	a	c	b	d
55	Linalyl butanoate	<0.01	c	b	a	b
56	β-Copaene	<0.01	a	b	b	b
57	*trans*-α-Bergamotene	<0.01	a	b	b	c
58	Aromadendrene	<0.01	a	b	c	c
59	epi-β-Santalene	<0.01	a	b	b	b
60	α-Humulene	<0.01	a	c	b	d
61	(*E*)-β-Farnesene	<0.01	c	a	c	b
62	9-epi-(*E*)-Caryophyllene	<0.01	b	b	a	b
63	Linalyl isovalerato	<0.01	b	a	a	a
64	Dauca-5,8-diene	<0.01	c	b	a	c
65	γ-Muurolene	<0.01	a	c	b	c
66	α-Amorphene	<0.01	a	b	b	b
67	*trans*-Muurola-4(14),5-	<0.01	a	b	c	c
68	(*E*)-Methyl Isoeugenol	<0.05	b	a	b	b
69	(*Z*)-α-Bisabolene	<0.05	b	a	b	ab
70	Lavandulyl isovalerate	<0.01	d	a	c	b
71	γ-Cadinene	<0.01	c	b	a	c
72	6-methyl-α-Ionone	<0.01	a	c	b	c
73	δ-Cadinene	<0.01	b	b	a	c
74	Spathulenol	<0.01	b	b	a	b
75	Caryophyllene oxide	<0.01	a	c	b	c
76	Guaiol	<0.01	b	b	a	b
77	1,10-di-epi-Cubenol	<0.01	b	b	a	b
78	α-Muurolol	<0.01	b	b	a	b
79	Bisabolol oxide B	<0.01	d	b	a	c
80	Helifolenol A	<0.01	a	c	b	c
81	α-Bisabolol	<0.01	d	b	a	c

For each compound, different letters for the Duncan test represent groups that are significantly different from each other using ANOVA at least p <0.05 (column 3); NS: not significant.
